# Wrist-worn optical and chest strap heart rate comparison in a heterogeneous sample of healthy individuals and in coronary artery disease patients

**DOI:** 10.1186/s13102-018-0098-0

**Published:** 2018-05-31

**Authors:** Francesco Sartor, Jos Gelissen, Ralph van Dinther, David Roovers, Gabriele B. Papini, Giuseppe Coppola

**Affiliations:** 1 Department of Personal Health, Philips Research, High Tech Campus 34, p.005, P.O. Box WB61, 5656 AE Eindhoven, The Netherlands; 2 Standardization Research & Robust Sensing, Philips Research, Eindhoven, The Netherlands; 3 Connected Sensing, Philips Patient Care & Monitoring Solutions, Eindhoven, The Netherlands; 4 Department of Electrical Engineering, Eindhoven University of Technology, Eindhoven, The Netherlands

**Keywords:** Heart rate, PPG, Evaluation, Accuracy

## Abstract

**Background:**

The need for unobtrusive HR (heart rate) monitoring has led to the development of a new generation of strapless HR monitors. The aim of this study was to determine whether such an unobtrusive, wrist-worn optical HR monitor (OHRM) could be equivalent and therefore a valid alternative to a traditional chest strap during a broad range of activities in a heterogeneous healthy population and coronary artery disease (CAD) patients.

**Methods:**

One hundred ninety-nine healthy volunteers, 84 males and 115 females, including 35 overweight-obese subjects, 53 pregnant women, and 20 CAD patients were tested in the present study. Second-by-second HR measured by the OHRM was concurrently evaluated against an ECG-based chest strap monitor during a broad range of activities (i.e., walking, running, cycling, gym, household, and sedentary activities).

**Results:**

Data coverage, percentage of time the OHRM provides a HR not larger than 10 bpm from the reference, went from a minimum of 92% of the time in the least periodic activity (i.e., gym), to 95% during the most intense activity (i.e., running), and to a maximum of 98% for sedentary activities. The limits of agreement of the difference between the OHRM and the chest strap HR were within the range of ±15 bpm. The OHRM showed a concordance correlation coefficient of 0.98. Overall, the mean absolute error was not larger than 3 bpm, which can be considered clinically acceptable for a number of applications. A similar performance was found for CAD (94.2% coverage, 2.4 bpm error), but the small sample size does not allow any quantitative comparison.

**Conclusion:**

Heart rate measured by OHRM at the wrist and ECG-based HR measured via a traditional chest strap are acceptably close in a broad range of activities in a heterogeneous, healthy population, and showed initial promising results also in CAD patients.

**Electronic supplementary material:**

The online version of this article (10.1186/s13102-018-0098-0) contains supplementary material, which is available to authorized users.

## Background

Heart rate (HR) is an important physiological parameter generated by the spontaneous activity of the sinoatrial pacemaker cells [1] and is chronically toned down by vagal activity [2]. It indicates the rhythm at which the heart pumps venous blood into the lungs and oxygenated blood into the systemic circulation, and as such reflects the metabolic need of the body. Because of this inherently vital central function, HR is influenced by a multitude of physiological and behavioral stimuli, such as: blood pressure, respiration, apnea, central motor command, posture, pain, water immersion, arousals, emotions and mechanical, physiochemical and biochemical metabolic changes [3]. As described by the Fick principle, HR is directly related to oxygen consumption [4]. This relation has been exploited to estimate cardiorespiratory fitness, also called VO_2max_, and energy expenditure [5, 6]. Yet, one of the most common applications of HR monitoring is to be found in sports, where HR is used to monitor exercise intensity [6].

In the 1980s, the growing need for HR monitoring in the sport industry market led to the development of wireless, wearable HR monitoring devices consisting of dry electrodes mounted on a chest strap sensor and a wristwatch radio receiver [6, 7]. They have been extensively validated in a large range of activities showing high overall accuracy (i.e., concordance correlation coefficient = 0.99; limits of agreement, LoA ± 2 bpm; ICC = 0.99) [8–11]. These chest strap HR monitors, although much more convenient than an electrocardiogram (ECG) Holter monitor [12], were still perceived as cumbersome and not ideal for everyday home monitoring, particularly in non-athletic persons [13, 14]. In the last decade, the need for less obtrusive HR monitoring led to the development of a new generation of strapless HR monitors, most of which are based on photoplethysmography (PPG) [15]. This technology provides a measure of the pulse-volume wave and consequently permits computation of the pulse rate. The main technical challenge of this method was, and still is, to effectively filter out the motion artifacts that pollute the PPG signal [15, 16]. Motion artifacts are distortions of the PPG signal as a result of subject-sensor movements. Cleaning the PPG signal of these artifacts becomes even more challenging when the relevant activities include periodic, quasi-periodic and non-periodic motion artifacts [17]. It is similarly challenging when, for the user’s convenience, the location chosen for the PPG sensor is at the medial part of the dorsal wrist [18]. This is due to several reasons, such as high, often erratic, motion levels experienced during daily activities as well as sports activities, the anatomical shape of the wrist (e.g., squared, conical, bony, rotund), which may not always fit well with the mechanical design of the device, and the micromotion generated by extensor digitorum tendons. All these affect sensor-skin optimal contact.

Although it is clear that this new, unobtrusive HR monitoring technology “could assist in the health-enhancing process of exercise or monitor cardiac and metabolic functions,” it must ensure an adequate level of reliability, verified by “evidence-based marketing claims” [19]. It is also important to explicitly define what is meant here for accurate HR. An acceptable error can range from about 2% at maximal levels, to 10% in resting conditions, depending on the use case [20, 21]. Consistently, the American National Standard of “Cardiac monitors, heart rate meters, and alarms” defines accuracy as a “readout error of no greater than ±10% of the input rate or ±5 bpm, whichever is greater” [22]. Currently, the accuracy of HR based on PPG sensors located at the wrist has been tested in healthy people in laboratory settings by several research teams, albeit resulting in an inconclusive opinion. Valenti and Westerterp [15], Delgado-Gonzalo et al. [23], Spierer et al. [7], and Wallen et al. [24] have reached a positive conclusion on the accuracy of PPG-based HR for activities ranging from a low wrist-motion level (e.g., standing, stationary cycling) to a high periodic motion level (e.g., walking, running). Conversely, Parak and Korhonen [13], Wang et al. [8], Gillinov et al. [25], and Cadmus-Bertram et al. [26] did not find PPG-based HR monitoring accurate enough for the same types of activities. This discrepancy in the literature can be explained in several ways. For one thing, different PPG sensor technologies were evaluated across these validation studies (i.e., Philips OHRM, Mio Alpha, Mio Link, Mio Fuse, Scosche Rhytm, Pulse On, Omron HR500U, Fitbit Charge HR, Samsung Gear S, Apple iWatch, Basis Peak). For another, sample sizes were rather variable across studies (from 19 to 50 subjects), impacting on the data variance and consequently on the standard errors. Moreover, methodological differences and limitations concerning laboratory protocols, data synchronization and data analysis contributed to these rather incoherent conclusions. Certainly, it is hard to achieve uniformity in validating HR monitoring derived from PPG sensor technology when all these variables play a role.

There is still scarce evidence of the reliability of wrist-worn PPG-based HR monitoring in patients. We have recently highlighted the clinical value of unobtrusive, continuous HR monitoring in cardiac patients. This encompasses diagnostic applications, such as exertion-induced tachycardia, detection of bradycardia, HR linked to symptoms, chronotropic incompetence, as well as therapeutic monitoring applications, such as beta-blockers titration (Sartor F, Papini, GB, Cox L, Cleland JFG: Methodological shortcomings of wrist-worn heart rate monitors validations, submitted). Recently, two non-wearable, PPG-based HR contact and contactless mobile applications were compared in 108 patients admitted at the chest pain unit or the emergency room [27]. Contactless PPG performed worse than contact finger PPG. The limitation of these non-wearables is that HR could be measured only at rest in absence of motion. Finally, a commercially available wrist-worn PPG watch (Fitbit Charge HR) has been recently tested in 50 intensive care unit patients, for whom the HR value was recorded every 5 m for 24 h compared to a bedside medical monitor [28]. The authors concluded that the HR watch accuracy worn by these bed-rested patients was poor, and in any case, less accurate than a pulse oximeter [28]. Yet, accuracy of the wrist-worn PPG-based HR monitoring technology needs to be shown in physically active patients.

Since the training concept of Frequency, Intensity, Time, and, Type has been adopted and recommended by the American College of Sports Medicine (ACSM), exercising at a personalized target HR has become the standard practice [29]. A large body of evidence shows that virtually anybody without known contraindications can benefit from cardiorespiratory training [29]. Nonetheless, specific populations, such as sedentary overweight or obese people, pregnant women, and coronary artery disease (CAD) patients undergoing rehabilitation, are particularly encouraged to undergo cardiorespiratory training. The ACSM recommends that sedentary overweight-obese people should engage in regular, low-to-moderate exercise intensity at 30–40% of their HR reserve [29]. Healthy women with uncomplicated pregnancies are advised to engage in physical exercise activity in order to reduce their risk of gestational weight gain and gestational diabetes [30]. Also for them, target HR is used to determine exercise intensity [30]. According to the European Society of Cardiology’s guidelines, CAD patients undergoing rehabilitation should “accumulate at least 30 min/day, 5 days/week of moderate intensity physical activity or 15 min/day, 5 days/week of vigorous intensity physical activity (75 min/week), or a combination of both, performed in sessions with a duration of at least 10 min,” where moderate intensity corresponds to 40–59%, and vigorous activity corresponds to 60–84% of the HR reserve [31]. Thus, accurate and unobtrusive HR-monitoring can be crucial for training within a personalized safe zone [8].

It is therefore of paramount importance to validate this new, unobtrusive way of measuring HR at the wrist in a set of circumstances that are more likely to occur in real life and with the people for whom it is recommend. The heterogeneity of the sample is key in adding ecological weight to its evaluation. For these reasons we have compared the HR derived from an innovative wrist-worn optical heart rate monitor (OHRM) to HR measured by a commercially available ECG-based chest strap monitor during a broad range of activities (i.e., walking, running, cycling, gym, household, and sedentary activities), in healthy normal weight as well as overweight-obese individuals, pregnant women and CAD patients. Consequently, the aim of this study was to determine whether such unobtrusive, wrist-worn OHRM could be equivalent, on a second-by-second level, and therefore a valid alternative to a traditional ECG-based chest strap during a broad range of activities in this heterogeneous sample.

## Methods

### Participants

This evaluation study of the Philips Electronics wrist-worn OHRM versus a traditional chest strap HR monitor was designed as a combination of numerous independent data collections. This is because we routinely collected data from a rather heterogeneous population executing a variety of activity types in order to improve and validate our HR algorithm. The full list of activities is listed in the study design paragraph. All observational protocols involving healthy people described in this study were approved by the institutional ethical committee review board of Philips Research Eindhoven. The protocol including CAD patients was approved by the local Medical Ethical Committee of Maxima Medical Center, Veldhoven. Both protocol approvals were obtained in conformity with the Declaration of Helsinki. Volunteers were recruited via posters internally to the Royal Philips Electronics organization and via a recruitment agency, or in the case of the CAD patients, by rehabilitation nurses. Prior to participation, volunteers received an oral and written explanation of our study procedure and they all provided written, informed consent.

The health status of the apparently healthy volunteers was assessed by the ACSM Health/Fitness Facility Pre-participation Screening Questionnaire [29]. All participants were of white ethnicity. Skin tone was measured by means of the Fitzpatrick skin typing scale [32] in only 40 participants, because it was matter of investigation only in a few data collections. The measurements produced a mean score of 2 ± 0.64 (where 0–6 is skin type I, white pale, and 35+ is black).

### Study protocol

Eighteen activities were executed in this study and were clustered into six categories: walking, running, cycling, gym, household, and sedentary (Additional file 1: Table S1). Walking activities included treadmill (95C, Life Fitness® Rosemont, Vermont, USA) walking at 3 km · h^− 1^, 3 km · h^− 1^ with 10% inclination, 4.5 km · h^− 1^, 4.5 km · h^− 1^ with 5% inclination, 5.5 km · h^− 1^, and 6 km · h^− 1^, and self-paced outdoor walking. Similarly, running activities consisted of treadmill running, with speed ranging from 6.5 km · h^− 1^ to 16 km · h^− 1^, and self-paced outdoor running. Cycling was also performed indoors on an ergometer (95 T, Rosemont, Vermont, USA) at 50, 70, and 90 rpm, with a minimum load of 60 watts up to 200 watts, as well as outdoor self-paced cycling on city, racing and mountain bikes provided by the research team on separate occasions. The exercises classified as gym activities consisted of rowing (pm4, Concept 2® Morrisville) between 25 and 32 strokes per minute, stepping (step, Reebok® Canton, Massachusetts, USA, height: 20 cm) at 96 bpm, cross-training (95X, Live Fitness® Rosemont, Vermont, USA) self-paced, and a series of group fitness activities such as Zumba, Tae-Bo, yoga/Pilates and boxing (punching bag). Household activities included vacuuming, cleaning the table, dishwashing, stacking groceries and deskwork (e.g., typing and writing by hand), and were conducted in our laboratories. All activities where little motion was present, such as resting and sitting, were clustered as sedentary types of activity. Because this database was built with data coming from multiple data collections as shown in the Additional file 1: Table S1, some subjects are present more than one time in the overall database.

Manual annotation derived by the predefined protocol was complemented with off-line analysis of the accelerometry data in order to accurately determine the start and the end of each activity. Typically, activities lasted 3 m, except for outdoor and group fitness activities, which lasted about 1 h. Indoor activities had rest intervals in a seated position between activities in order to lower the HR before starting a new activity. This allowed us to appreciate the HR rise and recovery per each single activity (Additional file 2: Figure S1).

### Data acquisition and experimental setup

Subjects were asked to wear an OHRM sensor built into a watch-like device with a silicon wrist strap (prototype developed by Philips Research), on one of their wrists regardless of their handedness (Additional file 2: Figure S1). This prototype is an evolution of the one previously described by Valenti and Westerterp [15]. They, however, tested only the first-generation PPG OHRM sensor, which had not yet been implemented in a wrist strap design. The wrist strap was positioned on the dorsal side of the wrist proximal to the ulnar process, and was strapped tight enough to limit its motion relative to the wrist without being uncomfortable for the wearer. This was done under supervision of a member of the research team. The OHRM prototype logged the PPG data (16, 32, 64 or 128 Hz) and the 3-axial accelerometer data (16 or 128 Hz). The real-time HR computation was based on a five-second sliding window and was used for the statistical analysis. The lower sample rates were introduced to reduce storage and to increase battery life for some applications. In addition, the real-time, OHRM-estimated HR together with a HR quality index were logged every second (1 Hz). The HR quality index was defined as an integer ranging from 0 (bad quality) to 4 (good quality) associated with the estimated HR and determined by the algorithm. This index represents an assessment step of the algorithm of the validity of its output. The data were stored in the internal memory of the prototype. These data were transferred via USB onto a personal computer at the end of each test. As the comparator, we used an ECG-based chest strap (H3, Polar Electro, Kempele, Finland) radio connected to a logging watch (RS800CX, Polar Electro, Kempele, Finland). The chest strap was set to output a HR value every second, except for data collections longer than 5 h, where it was set to output a HR value every 5 s.

### Data synchronization

Data were processed and analyzed using Matlab (Mathworks, Cambridge, MA, USA). The HR data from the OHRM and the chest strap were synchronized using an automated process in Matlab and by visual inspection. In the automated process the two sequences were interpolated on a uniform time grid by linear interpolation. The delay was calculated as the location of the maximum of the cross covariance function between the interpolated sequences, and the sequences were then aligned. A final visual inspection was performed to check the alignment and to discard erroneous reference data.

### Statistical analysis

Concurrent validity of the wrist-worn OHRM and the chest strap was evaluated using the following four metrics. *Availability* is defined as the percentage of time that the HR monitoring device is able to provide a heart rate measurement (quality > 0). *Coverage* represents the percentage of time that the OHRM prototype is able to provide a HR measurement (quality index > 0) and the measurement differs no more than 10 bpm from the reference measurement. *Mean absolute error* (MAE), *standard deviation of residuals or standard error of the estimate* (SEE), and *bias* were calculated for OHRM HR with a quality index > 0. The MAE and SEE are defined as follows:

1 $$ \mathrm{MAE}:= \frac{1}{L}{\sum}_{i=1}^L\left|{z}_i\right| $$

and

2 $$ \mathrm{SEE}:= \sqrt{\frac{\sum_{i=1}^L{\left({z}_i-\frac{1}{L}{\sum}_{j=1}^L{z}_j\right)}^2}{L-1}} $$

where *z*_*i*_ = *x*_*i*_ − *y*_*i*_, with *x*_*i*_ the output of the OHRM device and *y*_*i*_ the output of the reference device at time index *i*. The set *z*_1_, …, *z*_*L*_ consists of elements for which the OHRM device is able to provide a HR measurement (quality index > 0).

Furthermore, Bland-Altman plots and frequency distribution histograms were made using second-by-second HR values. Limits of agreement were calculated as the 95% confidence interval of the residuals. Additionally, a heat map (from blue to red) was used to visualize mean and residual HR data density.

## Results

One hundred nighty-nine volunteers (84 males, 115 females) were recruited and tested, including 53 healthy pregnant women and 20 CAD patients (Additional file 3: Table S2). In total, 371 h of data were collected, containing 25% cycling data, 18% gym data, 18% household data, 13% sedentary data, and 12 and 13% running and walking data, respectively (Table 1). The Additional file 2: Figure S1 shows HR data from a typical data collection including stepping, walking, cycling, cross-trainer walking, rowing, bicep curls and running with sedentary periods in between the activities. The highest coverage, as defined in the methods section, was found for sedentary activities (98.4%), and the lowest was found for gym activities (92.2%) (Table 1). Household activities produced the lowest mean HR (77.4 ± 13.8 bpm). Mean HR values in ascending order were 92.7 ± 25.5 bpm during sedentary activities, 98.2 ± 17.4 bpm for walking, 115.8 ± 23.1 bpm for cycling, 118.8 ± 30.1 bpm for gym activities and 143.2 ± 26.5 bpm for running. Mean absolute errors and SEEs did not exceed 3 and 10 bpm, respectively. All activities tested in this study showed a mean bias close to zero, excluding the possibility of systematic over- or under-estimation. This was statistically confirmed by the lack of significant heteroscedasticity (Table 1).

**Table 1:** Statistical evaluation of OHRM versus the chest-strap HR monitor

Activities	Hours collected	Coverage Δ ≤ 10 bpm (%)	Availability quality index > 0 (%)	MAE (bpm)	SEE (bpm)	Bias (bpm)
Healthy normal weight, BMI < 25						
Walking	20.6	95.4	99.2	1.8	4.7	− 0.2
Running	7.1	96.0	99.5	1.7	6.6	− 0.7
Cycling	11.3	98.4	99.9	0.8	2.6	−0.3
Gym	60.6	92.5	98.6	2.9	9.3	−1.1
Household	–	–	–	–	–	–
Sedentary	21.0	98.0	99.9	1.3	5.5	−0.3
Overall	120.5	94.7	99.1	2.2	7.4	−0.7
Healthy over-weight, BMI > 25						
Walking	6.4	97.1	99.5	1.2	3.2	0.1
Running	2.0	98.8	99.9	0.8	2.8	−0.3
Cycling	3.8	98.9	99.9	0.7	2.2	−0.2
Gym	22.6	95.6	99.3	2.0	7.0	−0.7
Household	–	–	–	–	–	–
Sedentary	3.4	99.3	100.0	0.9	3.9	−0.1
Overall	38.2	96.6	99.5	1.5	5.8	−0.5
Pregnant						
Walking	9.6	93.7	98.9	2.4	6.1	−0.1
Running	–	–	–	–	–	–
Cycling	3.4	99.9	100.0	0.3	1.0	0.1
Gym	3.4	86.4	97.6	3.4	7.2	−2.0
Household	9.6	86.0	98.4	4.2	7.3	0.7
Sedentary	1.7	97.9	100.0	1.1	2.7	−0.6
Overall	27.7	91.1	98.8	2.8	6.2	−0.1
CAD						
Walking	2.6	96.9	99.9	1.7	4.2	1.0
Running	–	–	–	–	–	–
Cycling	2.1	85.6	98.5	4.4	7.1	2.6
Gym	0.8	95.1	99.0	2.7	4.2	0.7
Household	3.5	95.2	99.4	2.2	4.9	0.4
Sedentary	1.4	99.7	100.0	0.8	1.6	0.1
Overall	10.3	94.2	99.4	2.4	5.0	1.0
All						
Walking	49.9	94.6	99.0	2.0	5.2	−0.0
Running	43.6	95.2	99.0	2.0	7.2	−0.5
Cycling	93.4	96.0	99.5	1.7	5.3	−0.4
Gym	67.5	92.2	98.6	3.0	9.5	−1.2
Household	67.6	94.7	99.6	2.3	5.0	−0.4
Sedentary	49.0	98.4	99.8	1.2	5.7	−0.4
Overall	371.1	95.1	99.3	1.4	6.5	−0.5

*OHRM* Optical Heart Rate Monitor, *MAE* Mean Absolute Error, *SEE* Standard Error of the Estimate, *CAD* Coronary Artery Disease

**Fig. 1:**
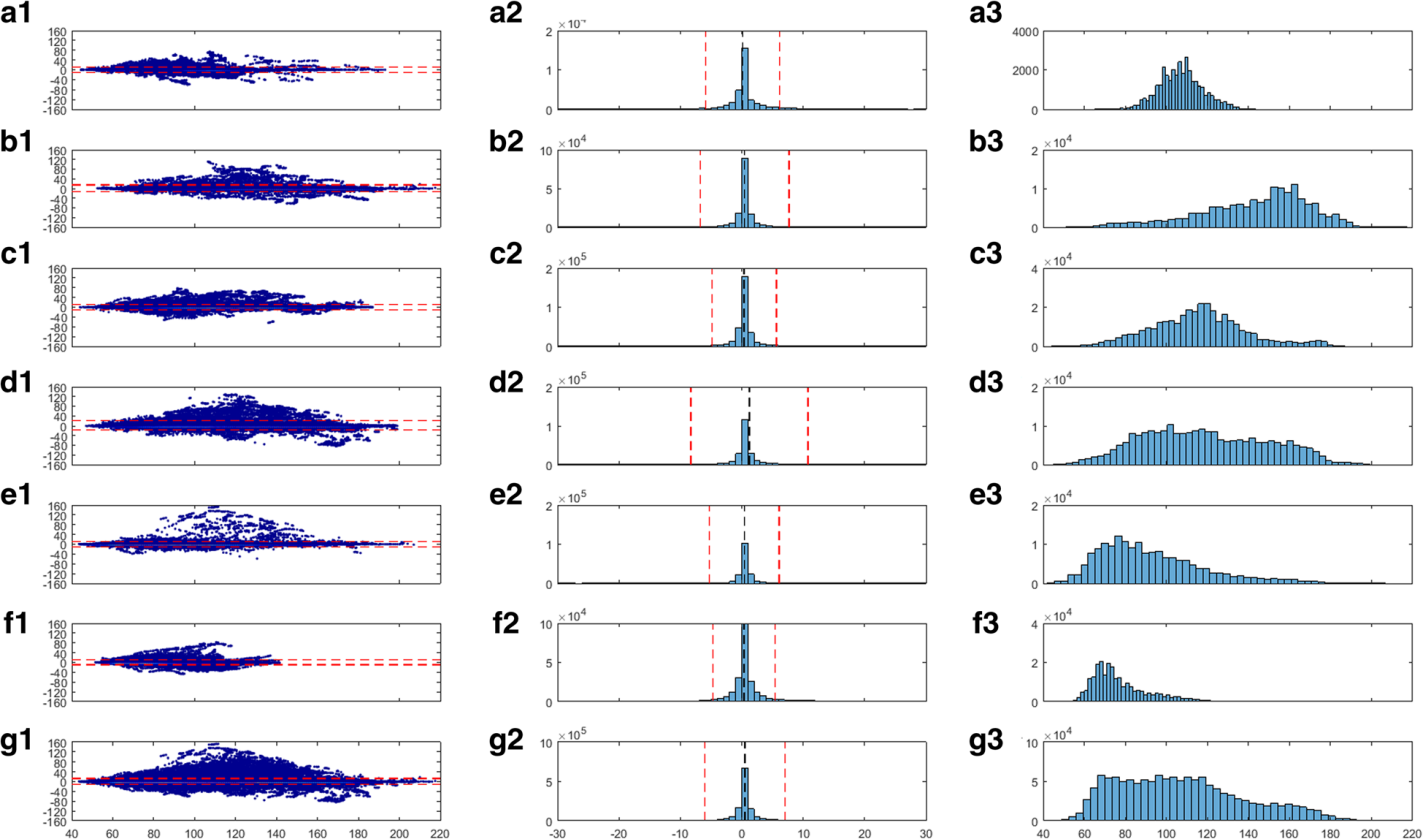
A1) Residuals against means of chest strap electrocardiogram (ECG)-based heart rate (HR) and wrist strap photoplethysmography (PPG)-based HR for walking, where the red dashed lines represent 95% confidence intervals. Color coding (blue = low to red = high) highlights the peak data density for both residuals and means. A2) shows the frequency distribution of the difference between chest strap ECG and wrist-strap PPG; red dashed lines represent plus, minus 1 standard deviation, and the black dashed line represents the mean difference. A3) shows the frequency distribution of the mean HR measured by the two methods. B1,2,3) for running; C1,2,3) for cycling; D1,2,3) for gym activities; E1,2,3) for sedentary activities; F1,2,3) for household activities and G1,2,3) for all activities

The distribution histograms reported in the Fig. 1 show that biases had standard deviations below 10 bpm, meaning that the large majority of residual data points did not deviate from the zero bias. This was also highlighted by the heat maps, which located the overall data density around the zero bias line. The heterogeneity of the individuals and activities tested generated a broad range of HR values ranging from 40 to 220 bpm, with a non-Gaussian, platykurtic distribution bi-modally skewed at 70 bpm and 120 bpm. Overall the LoA of the difference between the OHRM and the chest strap HR were below 15 bpm, namely between − 12.3 bpm and 13.3 bpm. For locomotor activities (running and walking), the LoA were between − 13.7 bpm and 14.6 bpm and between − 10.2 bpm and 10.3 bpm, respectively. Similarly, for cycling, LoA were between − 9.9 bpm and 10.7 bpm. The largest LoA (− 17.5 bpm and 19.9 bpm) were found for gym activities, which is the most diverse set of physical activities. Low-intensity activities showed also the lowest LoA, with − 9.5 bpm and 10.3 bpm for household activities, and slightly higher values for sedentary activities − 10.8 bpm and 11.5 bpm.

The Table 1 shows separate statistics for the healthy normal weight, healthy overweight, pregnant women and CAD patients included in the database.

## Discussion

The primary outcome of this study is that HR measured at the wrist by a PPG-based OHRM can be used in a very heterogeneous population including overweight/obese people pregnant women, and CAD patients during a large range of activities such as locomotor, gym and household activities, where the OHRM performs comparably well as a traditional chest strap ECG-based measuring device.

### Data coverage

To the authors’ knowledge, this is the largest published second-by-second comparison (371 h) between wrist-worn PPG-based HR and chest strap ECG-based HR. Data coverage did not fall below 92.2%, even in unpredictable non-periodic activities such as gym activities, which included Zumba, boxing, and Tae Bo, activities that had major impacts on the wrist where the OHRM was placed. Consistently, highly periodic activities, which are typically known to a have high level of motion artifacts, such as running [33], showed a higher data coverage in this study than less periodic activities, such as gym and household activities. Encouragingly, data coverage was still rather high for household activities (94.7%), which covered a diverse type of physical activities. Not surprisingly, the highest data coverage was found in activities with the lowest effect of motion artifacts, such as cycling and sedentary activities. Yet, it is hard to compare our coverage data with the existing literature data in general because of a lack of uniformity in methodology and reporting. Valenti and Westerterp [15] validated the first generation OHRM, of which the latest version is considered in the present study. Consistent with the present study, they have evaluated HR accuracy on one-second epochs and used the same definition of data coverage [15]. They reported an overall data coverage of 86% on the entire protocol, which included resting, walking and running up to 20 km/h [15]. Interestingly their mean coverage for running was around 74%, ranging from 55% at 16 km/h to 85% at 9 km/h [15]. The mean coverage for running in the present study, where the latest OHRM generation was used, was far higher (95.2%) than in Valenti and Westerterp [15], showing a considerable improvement. This enhancement can be ascribed to improvements in the optical unit as well as the HR extraction algorithm. Parak & Korhonen [13] and Delgado-Gonzalo et al. [23] used five-second averages and reported an overall data coverage of around 86% for two PPG-based monitors, Mio Alpha and Scosche myRhythm, and 87% for Mio Link and 94% for PulseOn in a protocol that included resting, walking, running, and cycling. A very high coverage (99%) was reported for running with respect to the PulseOn and the Mio Link [23]. Yet in these studies, data were averaged each 5 s, thus smoothing part of the HR signals. Unfortunately, more recent studies did not provide this type of information and were methodologically limited by the manual collection of the HR values [8, 24].

### Mean and standard errors

The largest mean absolute and standard errors of the OHRM were observed mainly in activities containing high levels of non-periodic wrist movements, such as household and gym. These were in any case not greater than 2.3 and 3 bpm, respectively. During locomotor activities the MAE and SEE were below 2 and 8 bpm, respectively. In general, the MAEs of the OHRM in the present study were lower than those reported in literature. Spierer et al. [7] reported a MAE of 2.4 bpm at rest, 2.4 bpm during walking, 5 bpm during running, and 3.3 bpm for cycling for the Mio Alpha and reported 2.2 bpm at rest, 5 bpm during walking, 6 bpm during running, with the exception of 1 bpm during cycling, for the HR500U. Furthermore, Parak and Korhonen [13] observed a MAE of 4 bpm at rest, 5 bpm during walking, 2.9 bpm during running, and 4.6 bpm during cycling for the Mio Alpha, and recorded 4.8 bpm at rest, 10.5 bpm during walking, 6.7 bpm during running, and 1.8 bpm during cycling for the Scosche MyRhythm. Still, it is important to point out that errors were reported as minute averages in Spierer et al. [7] and as five-second averages in Parak and Korhonen [13]. Nevertheless, comparing MAE and SEE is not straightforward because of the differences in averaging and outlier filtering strategies.

### Limits of agreement

Limits of agreement in the present study were calculated on all data with no outlier rejection, and were found below ±15 bpm (see Table 1). This is an excellent result when considering the number of subjects, activities and second-by-second analysis. In the literature, LoA of other optical wrist-worn HR devices are generally poorer. In Wallen et al. [24], who tested the Apple Watch, Fitbit Charge, Samsung Gear S and Mio Alpha in 22 healthy subjects, LoA ranged from − 27 bpm to + 13 bpm for running and cycling on a cycle ergometer, and in Wang et al. [8], who tested 50 healthy subjects, LoA ranged from − 27 bpm to + 29 bpm for walking and running on the treadmill using the Apple Watch and Mio Fuse. Gillinov et al. [25] tested four wrist-worn watches and one commercially-available armband in 50 healthy subjects. They found LoA from − 17 bpm to 20 bpm for the Apple Watch, − 24 bpm to 31 bpm for TomTom Spark Cardio, − 24 bpm and 31 bpm for Garmin Forerunner 235, − 31 bpm to 38 bpm for Scosche Rhythm, and − 30 bpm to 45 bpm for Fitbit Blaze. Their research protocol including treadmill walking and running, stationary bike cycling and elliptical trainer exercise. Nevertheless, Cadmus-Bertram et al. [26] found LoA smaller than in our study (±11.5 bpm) during resting, in 40 healthy subjects. The LoA values were from − 4.1 bpm to 4.5 bpm for Surge Fitbit, − 17.1 bpm to 22.6 bpm for Basis Peak, − 10.5 bpm to 4.5 bpm for Fitbit Charge, and − 7.8 bpm to 9.9 bpm for Mio Fuse. At the same time, poor LoA were found for 65% of HR_max_ treadmill running, with values ranging from − 34.8 bpm to 39.0 bpm for Surge Fitbit, − 27.1 bpm to 29.2 bpm for Basis Peak, − 41.0 bpm to 36.0 bpm for Fitbit Charge, and − 22.5 bpm to 26.0 bpm for Mio Fuse.

Although these four studies employed basic and controlled laboratory protocols and a smaller sample size than our study, the LoA they reported were much larger than those observed in the present study. An exception to this was LoA values for Surge and Charge Fitbit ate rest when compared with our sedentary activity values. Furthermore, it is important to note that in Wallen et al. [24] and Wang et al. [8], as well as in Gillinov et al. [25] and Cadmus-Bertram et al. [26], the HR values were gathered manually, whereas in the present study Bland-Altman plots were built on a second-by-second basis.

### General accuracy

Given the large spectrum of applications where HR monitoring can be used, as described in the introduction, the required accuracy will depend upon each specific case. Wang et al. and Gillinov et al. [8, 25] used a 0.95 concordance correlation coefficient as the accuracy threshold. The concordance correlation coefficient takes into account the deviation of the observations from the line of best fit and the deviation of this from the ideal trend line. In the present study, we have calculated an overall concordance correlation coefficient in the entire database of 0.977, which corresponds to a good accuracy of the OHRM. Already in 1977, Cumming & Glenn [34] showed how an error in HR measurement would lead to a wrong estimation in cardiorespiratory fitness (e.g., a 12 bpm error would lead to an error of 25% in prediction of cardiorespiratory fitness). Moreover, more recently it was shown how error tolerance depends on the purpose of the measurement [20]. For instance, the error may not exceed 3 bpm to measure a maximum HR, and it should remain below 8 bpm to prescribe a training target HR [20]. Consistently, in atrial fibrillation, an error of 10% (around 12 bpm) was used as the criterion for clinical significance [21]. More recently the American National Standard of “Cardiac monitors, heart rate meters, and alarms” has defined accuracy as: “readout error of no greater than ±10% of the input rate or ±5 bpm, whichever is greater” [22].

Taken together, these observations show that the acceptable error can range from about 2 to 10% depending on the use case. In this study the PPG-based OHRM measured HRs with a MAE and SEE of at most 3 bpm (~ 2.5%) and 10 bpm (8%), which is clinically acceptable for a number of applications based on the aforementioned information.

Although only 3 and 7% of the collected data came from male CAD patients and pregnant women respectively, it can be appreciated on a qualitative basis that, for walking and sedentary activities, coverage accuracy was in line with the overall results. Whereas CAD patients seemed to have a slightly lower coverage and higher errors for cycling, pregnant women showed slightly worse coverage and accuracy for gym activities. Household and sedentary activities were in line with the overall results.

The main strength of this study is its large and diverse HR database. Moreover, the comparison of HR values was based on second-by-second synchronized data. This study also has several limitations. First of all, raw ECG traces were not used as a reference. Instead, we relied on a chest strap HR monitor. This type of monitor has been shown to be highly reliable in measuring HR in a broad range of activities. However, it could be less accurate in activities where important levels of chest movement are performed (e.g., yoga/Pilates). This could have been objectively assessed with a complete ECG trace. Moreover, in this study we did not include any other device, thus not allowing a direct comparison to be made, but only for comparisons from the literature. Although, we have included 20 CAD patients in this analysis, this technology needs to be extensively validated in patient populations. The performance of the OHRM has been shown elsewhere for atrial fibrillation patients [35]. Future research should validate this unobtrusive HR monitoring technology in larger patient populations, such as cardiac patients on HR-lowering medications undergoing cardiac rehabilitation exercise. Because of the nature of this dataset, which included more data collections, some subjects have taken part in more of the experiments. This may have reduced the variance of the data to some extent. However, there was no systematic selection of “good” or “bad” subjects with respect to the PPG signal. Therefore, the impact of the multiple participations in the study by some of the subjects should be minimal.

## Conclusion

The HR measured by the wrist-worn PPG-based OHRM is acceptably close to the HR measured by a traditional ECG-based chest strap. The error was at most 3 bpm, and the LoA were smaller than 15 bpm. OHRM and chest strap HR monitoring could be mutually used in a broad variety of physical activities, such as locomotor, cycling, gym, household and sedentary activities in apparently healthy normal-weight as well as overweight/obese persons and pregnant women. Data coverage went from a minimum of 92.2% of the time in the least periodic activity (i.e., gym), to 95.2% during the most intense activity (i.e., running), and to a maximum of 98.4% during sedentary activities. These results imply that the PPG-based HR could be used with a satisfactory level of accuracy in a wide variety of healthy people. Positive preliminary evidence was also provided here for CAD patients. Future research should focus on proof-of-concept studies in target populations (e.g., rehabilitation).

## Additional files

Additional file 1: **Table S1.** Database overview. (DOCX 16 kb)

Additional file 2: **Figure S1.** A) Heart rate (HR) data for a representative subject during a mixed protocol. Green stars represent Optical Heart Rate Module HR values, and the purple line represents the chest strap HR reference. B) Photographs of the Optical Heart Rate Module mounted on a wrist strap. (TIF 427 kb)

Additional file 3: **Table S2.** Subjects characteristics. (DOCX 15 kb)

## References

[1] Keith A, Flack M (1907). The form and nature of the muscular connections between the primary divisions of the vertebrate heart. J Anat Physiol.

[2] Patterson SW, Piper H, Starling EH (1914). The regulation of the heart beat. J Physiol.

[3] Abboud FM, Thames MD, Pollock DM (1983). Interaction of cardiovascular reflexes in circulatory control. Comprehensive physiology.

[4] Fick AE (1870). On the measurement of the blood quantity in the ventricles of the heart.

[5] Sartor F, Vernillo G, de Morree HM, Bonomi AG, La Torre A, Kubis HP (2013). Estimation of maximal oxygen uptake via submaximal exercise testing in sports, clinical, and home settings. Sports Med.

[6] Achten J, Jeukendrup AE (2003). Heart rate monitoring: applications and limitations. Sports Med.

[7] Spierer DK, Rosen Z, Litman LL, Fujii K (2015). Validation of photoplethysmography as a method to detect heart rate during rest and exercise. J Med Eng Technol.

[8] Wang R, Blackburn G, Desai M, Phelan D, Gillinov L, Houghtaling P (2016). Accuracy of wrist-worn heart rate monitors. JAMA Cardiol.

[9] Weippert M, Kumar M, Kreuzfeld S, Arndt D, Rieger A, Stoll R (2010). Comparison of three mobile devices for measuring R-R intervals and heart rate variability: polar S810i, Suunto t6 and an ambulatory ECG system. Eur J Appl Physiol.

[10] Kingsley M, Lewis MJ, Marson RE (2005). Comparison of polar 810s and an ambulatory ECG system for RR interval measurement during progressive exercise. Int J Sports Med.

[11] Laukkanen RM, Virtanen PK (1998). Heart rate monitors: state of the art. J Sports Sci.

[12] Holter NJ (1961). New method for heart studies. Science.

[13] Parak J, Korhonen I. Evaluation of wearable consumer heart rate monitors based on photopletysmography. Conference proceedings: Annual International Conference of the IEEE Engineering in Medicine and Biology Society IEEE Engineering in Medicine and Biology Society Annual Conference. 2014;2014:3670–3673. 10.1109/EMBC.2014.6944419 25570787

[14] Andre D, Wolf DL (2007). Recent advances in free-living physical activity monitoring: a review. J Diab Sci Technol.

[15] Valenti G, Westerterp KR. Optical heart rate monitoring module validation study. Ieee Icce. 2013:195–6.

[16] Allen J (2007). Photoplethysmography and its application in clinical physiological measurement. Physiol Meas.

[17] Wijshoff R, Mischi M, Aarts R (2016). Reduction of periodic motion artifacts in photoplethysmography. IEEE Trans Biomed Eng.

[18] Maeda Y, Sekine M, Tamura T (2011). Relationship between measurement site and motion artifacts in wearable reflected Photoplethysmography. J Med Syst.

[19] Sperlich B, Holmberg HC (2017). Wearable, yes, but able...?: it is time for evidence-based marketing claims!. Br J Sports Med.

[20] Robergs RA, Landwehr R (2002). The surprising history of the HRmax=220-age equation. J Exerc Physiol Online.

[21] Sneed NV, Hollerbach AD (1992). Accuracy of heart rate assessment in atrial fibrillation. Heart Lung.

[22] ANSI/AAMI (2002). Cardiac monitors, heart rate meters, and alarms.

[23] Delgado-Gonzalo R, Parak J, Tarniceriu A, Renevey P, Bertschi M, Korhonen I. Evaluation of accuracy and reliability of PulseOn optical heart rate monitoring device. Conference proceedings: Annual International Conference of the IEEE Engineering in Medicine and Biology Society IEEE Engineering in Medicine and Biology Society Annual Conference. 2015;2015:430–3. 10.1109/EMBC.2015.7318391 26736291

[24] Wallen MP, Gomersall SR, Keating SE, Wisloff U, Coombes JS (2016). Accuracy of heart rate watches: implications for weight management. PLoS One.

[25] Gillinov S, Etiwy M, Wang R, Blackburn G, Phelan D, Gillinov AM (2017). Variable accuracy of wearable heart rate monitors during aerobic exercise. Med Sci Sports Exerc.

[26] Cadmus-Bertram L, Gangnon R, Wirkus EJ, Thraen-Borowski KM, Gorzelitz-Liebhauser J (2017). The accuracy of heart rate monitoring by some wrist-worn activity trackers. Ann Intern Med.

[27] Coppetti T, Brauchlin A, Muggler S, Attinger-Toller A, Templin C, Schonrath F (2017). Accuracy of smartphone apps for heart rate measurement. Eur J Prev Cardiol.

[28] Kroll RR, Boyd JG, Maslove DM (2016). Accuracy of a wrist-worn wearable device for monitoring heart rates in hospital inpatients: a prospective observational study. J Med Internet Res.

[29] American College of Sports Medicine (2010). ACSM’s resource manual for guidelines for exercise testing and prescription.

[30] Zavorsky GS, Longo LD (2011). Exercise guidelines in pregnancy: new perspectives. Sports Med.

[31] Piepoli MF, Hoes AW, Agewall S, Albus C, Brotons C, Catapano AL (2016). 2016 European Guidelines on cardiovascular disease prevention in clinical practice: The Sixth Joint Task Force of the European Society of Cardiology and Other Societies on Cardiovascular Disease Prevention in Clinical Practice (constituted by representatives of 10 societies and by invited experts)Developed with the special contribution of the European Association for Cardiovascular Prevention & Rehabilitation (EACPR). Eur Heart J.

[32] Fitzpatrick TB (1988). The validity and practicality of sun-reactive skin types I through VI. Arch Dermatol.

[33] Han H, Kim J (2012). Artifacts in wearable photoplethysmographs during daily life motions and their reduction with least mean square based active noise cancellation method. Comput Biol Med.

[34] Cumming GR, Glenn J (1977). Evaluation of the Canadian home fitness test in middle-aged men. Can Med Assoc J.

[35] Bonomi AG, Schipper F, Eerikäinen LM, Margarito J, Aarts RM, Babaeizadeh S, et al. Atrial fibrillation detection using photo-plethysmography and acceleration data at the wrist. In: IEEE Engineering in Medicine and Biology Society, editor. Computing in cardiology; 2016 Sep 11–14; Vancouver (Canada) 2016.

