# Supplementary material for: Wrist-worn optical and chest strap heart rate comparison in a heterogeneous sample of healthy individuals and in coronary artery disease patients
Source: BMC Sports Sci Med Rehabil. 2018 May 31;10:10. doi: 10.1186/s13102-018-0098-0 (PMC5984393; doi:10.1186/s13102-018-0098-0)
Supplement: Supplementary file 1 — Table S1. Database overview. (DOCX 16 kb) [file 13102_2018_98_MOESM1_ESM.docx]

| **Additional file 1: Table S1** Database overview | | | |
| --- | --- | --- | --- |
| **Data collections** | **Activities** | **Hours** | **Number of tests** |
| 1 | Zumba, pilates/yoga, boxing, tae bo | 46.2 | 52 |
| 2 | Walking, cycling, running | 4.6 | 21 |
| 3 | Cycling^†^, running^†^ | 24.7 | 25 |
| 4 | Walking, cycling, running, cross-trainer, rowing, stepping | 18.3 | 19 |
| 5 | Walking, cycling, running | 2.9 | 6 |
| 6 | Sitting, walking, cycling, running, running^†^ | 2.8 | 4 |
| 7 | Walking, cycling, running, rowing, stepping | 30.6 | 42 |
| 8 | Sitting, walking, cycling, cycling^†^, running, running^†^ | 15.2 | 22 |
| 9 | Walking, cycling, running, rowing, stepping | 25.1 | 30 |
| 10 | Walking, cycling, running, cycling^†^, rowing, stepping | 5 | 8 |
| 11 | Walking, walking^†^, mountainbike cycling^†^, running | 15.6 | 19 |
| 12 | Desk work, sitting, household activities, leisure activities, cycling, running | 59.7 | 16 |
| 13 | Sitting, walking, cycling, running ,cross-trainer, bicep curls | 6.2 | 15 |
| 14 | Sitting, walking, running | 1.6 | 4 |
| 15 | Standing, standing^†^, walking, walking^†^, running, running^†^ | 2 | 5 |
| 16 | Sitting, walking, cycling, running | 7.7 | 12 |
| 17 | Sitting, walking, running, rowing | 3.1 | 6 |
| 18 | Sitting, walking, running | 2.3 | 6 |
| 19 | Cycling^†^ | 8.1 | 5 |
| 20 | Sitting, walking, walking^†^, cycling, cycling^†^, household activities | 27.3 | 34 |
| 21 | Sitting, walking, cycling, running, running^†^ | 10.2 | 26 |
| 22 | Sitting, walking, running | 2.2 | 4 |
| 23 | Sitting, walking, cycling, running, rowing, stepping | 1.9 | 3 |
| 24 | Sitting, walking, cycling, running, rowing, stepping | 4 | 5 |
| 25 | Cycling^†^ | 18.1 | 33 |
| 26 | Cycling^†^, e-bike cycling^†^ | 11 | 24 |
| 27 | Sitting, standing, walking, cycling, household activities | 11.4 | 17 |
| Total |  | 367.8 | 463 |

†Outdoors activities
