# Supplementary figures and images for: Wrist-worn optical and chest strap heart rate comparison in a heterogeneous sample of healthy individuals and in coronary artery disease patients
Source: BMC Sports Sci Med Rehabil. 2018 May 31;10:10. doi: 10.1186/s13102-018-0098-0 (PMC5984393; doi:10.1186/s13102-018-0098-0)

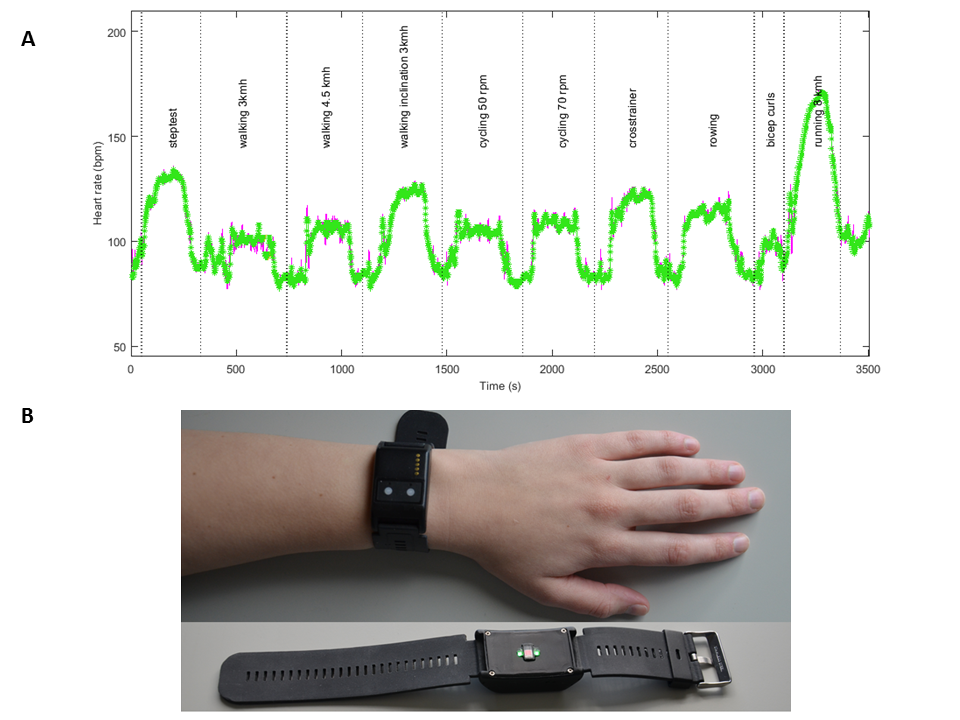

Supplement: Supplementary file 2 — Figure S1. A) Heart rate (HR) data for a representative subject during a mixed protocol. Green stars represent Optical Heart Rate Module HR values, and the purple line represents the chest strap HR reference. B) Photographs of the Optical Heart Rate Module mounted on a wrist strap. (TIF 427 kb) [file 13102_2018_98_MOESM2_ESM.tif]
