# Supplementary material for: Wrist-worn optical and chest strap heart rate comparison in a heterogeneous sample of healthy individuals and in coronary artery disease patients
Source: BMC Sports Sci Med Rehabil. 2018 May 31;10:10. doi: 10.1186/s13102-018-0098-0 (PMC5984393; doi:10.1186/s13102-018-0098-0)
Supplement: Supplementary file 3 — Table S2. Subjects characteristics. (DOCX 15 kb) [file 13102_2018_98_MOESM3_ESM.docx]

| **Additional file 3: Table S2** Subjects characteristics | | | | | |
| --- | --- | --- | --- | --- | --- |
|  | Healthy normal-weight BMI < 25  (n=91) | Healthy overweight-Obese  BMI > 25 (n=35) | Pregnant women (n=53) | CAD  (n=20) | All  (n = 199) |
| Males/Females | 43/48 | 22/13 | 0/53 | 19/1 | 84 ± 115 |
|  | mean ± SD | mean ± SD | mean ± SD | mean ± SD | mean ± SD |
| Age (y) | 33 ± 9 | 33 ± 10 | 30 ± 4 | 56 ± 7 | 38 ± 7 |
| Weight (kg) | 65.6 ± 9.1 | 83.1 ± 10.1 | 73.0 ± 13.7 | 93.1 ± 11.9 | 78 ± 11.2 |
| Height (m) | 1.74 ± 0.08 | 1.74 ± 0.09 | 1.69 ± 0.07 | 1.81 ± 0.07 | 1.74 ± 0.08 |
| BMI (kg/m^2^) | 21.6 ± 1.8 | 27.4 ± 1.8 | 25.7 ± 4.5 | 28.6 ± 4.1 | 25.8 ± 3.0 |
| SD, standard deviation; BMI, body mass index; CAD, coronary artery disease | | | | | |
